# Global N^6^-Methyladenosine Profiling Revealed the Tissue-Specific Epitranscriptomic Regulation of Rice Responses to Salt Stress

**DOI:** 10.3390/ijms23042091

**Published:** 2022-02-14

**Authors:** Yinxiao Wang, Fengping Du, Yingbo Li, Juan Wang, Xiuqin Zhao, Zhikang Li, Jianlong Xu, Wensheng Wang, Binying Fu

**Affiliations:** 1 Institute of Crop Sciences, National Key Facility for Crop Gene Resources and Genetic Improvement, Chinese Academy of Agricultural Sciences, Beijing 100081, China; hengshuiyin@163.com (Y.W.); 18643702970@163.com (F.D.); lyb156636865902021@163.com (Y.L.); wangjuan22@outlook.com (J.W.); zhaoxiuqin@caas.cn (X.Z.); lizhikang@caas.cn (Z.L.); xujianlong@caas.cn (J.X.); 2 Biotechnology Research Institute, Chinese Academy of Agricultural Sciences, Beijing 100081, China; 3 College of Agronomy, Anhui Agricultural University, Hefei 230036, China; 4 National Nanfan Research Institute (Sanya), Chinese Academy of Agricultural Sciences, Sanya 572024, China

**Keywords:** epitranscriptome, gene regulation, m^6^A methylation, MeRIP-seq, rice, salt stress

## Abstract

N^6^-methyladenosine (m^6^A) methylation represents a new layer of the epitranscriptomic regulation of plant development and growth. However, the effects of m^6^A on rice responses to environmental stimuli remain unclear. In this study, we performed a methylated-RNA immunoprecipitation sequencing analysis and compared the changes in m^6^A methylation and gene expression in rice under salt stress conditions. Salt stress significantly increased the m^6^A methylation in the shoots (*p* value < 0.05). Additionally, 2537 and 2304 differential m^6^A sites within 2134 and 1997 genes were identified in the shoots and roots, respectively, under salt stress and control conditions. These differential m^6^A sites were largely regulated in a tissue-specific manner. A unique set of genes encoding transcription factors, antioxidants, and auxin-responsive proteins had increased or decreased m^6^A methylation levels only in the shoots or roots under salt stress, implying m^6^A may mediate salt tolerance by regulating transcription, ROS homeostasis, and auxin signaling in a tissue-specific manner. Integrating analyses of m^6^A modifications and gene expression changes revealed that m^6^A changes regulate the expression of genes controlling plant growth, stress responses, and ion transport under saline conditions. These findings may help clarify the regulatory effects of m^6^A modifications on rice salt tolerance.

## 1. Introduction

RNA molecules, which have dual roles as information carriers and gene expression regulators, play crucial roles in cellular mechanisms affecting genetic messages, biochemical reactions, and the cell cytoskeleton in all living organisms [1]. RNA modifications are post-transcriptional regulatory processes that provide a biochemical basis for functional diversity. Of the more than 100 types of RNA modifications, the most important is N^6^-methyladenosine (m^6^A) in eukaryotic rRNA, tRNA, snRNA, and mRNA [2]. The m^6^A methylation, which is the most prevalent modification in mRNA, affects plant development and growth by regulating the processing, transport, stability, and translation of mRNA [3].

The m^6^A modification, which is a reversible biological process mediated by writers/methylases and erasers/demethylases, is recognized by readers/RNA-binding proteins [4]. Studies revealed that m^6^A methylation is a highly conserved mRNA modification in plants and animals. More specifically, m^6^A is generated by the consensus sequence RRACH (R is G or A; H is U, A, or C), and this modification is highly enriched near the stop codon or 3′ untranslated region (UTR) in plants and animals [5,6] as well as around the start codon in plants [7,8].

Recent gene functional studies regarding m^6^A writers, readers, and erasers highlighted an important role for m^6^A mRNA modifications in plant gene regulation during responses to abiotic stresses. Gene expression studies demonstrated that a few of these writers, erasers, and readers in *Arabidopsis thaliana* and rice are differentially modulated by diverse abiotic stresses [7,9,10]. The *A. thaliana* reader ECT2 affects the heat stress response by modifying mRNA stability and relocating to stress granules [11,12]. The knockdown of the RNA demethylase ALKBH6 in *A. thaliana* increases the sensitivity of plants to salt, drought, and heat stresses [13]. Overexpressing *PtrMTA* can enhance the drought tolerance of *Populus* species by altering trichome and root development [14]. The molecular mechanisms underlying the responses of these m^6^A-related genes to abiotic stress need to be more thoroughly investigated.

Global epitranscriptome studies revealed that m^6^A mRNA methylation in plants is highly sensitive to abiotic stresses, including drought, salt, and heat. Moreover, m^6^A methylation can increase the stability of salt-tolerance-related transcripts, thereby regulating salt tolerance in *A. thaliana* [15]. Further genome-wide m^6^A profiling using mutants of m^6^A writer components highlighted the importance of m^6^A methylation for *A. thaliana* salt tolerance. More specifically, m^6^A sites and 3′ UTR length are closely associated with transcript stability during adaptations to stress [16]. In various maize genotypes, the diversity in the extent of the m^6^A modifications of a unique set of transcripts influences the regulation of drought responses [17]. Additionally, the number and extent of m^6^A methylations of the mRNA of salt-resistance genes affect the salt tolerance of *A. thaliana* and sweet sorghum [18,19].

Rice is glycophytic in nature and highly sensitive to salt stress. High salinity stimulates many resistance-related responses, including morphological, physiological, and molecular changes in cells as well as epigenetic events [20,21,22]. The development of the methylated-RNA immunoprecipitation high-throughput sequencing (MeRIP-seq) platform enabled the transcriptome-wide m^6^A methylation profiling of two rice tissues, which revealed the tissue specificity of m^6^A methylation and the correlation between m^6^A peak enrichment and gene expression [7]. To date, there has been no report describing the m^6^A methylation related to rice responses to salt stress. In the present study, the MeRIP-seq platform was used to investigate the m^6^A methylation changes in response to salt stress in the shoots and roots of FL478, which is a good source of salt tolerance at the seedling stage in rice. Our results elucidated the tissue-specific and stress-modulated aspects of m^6^A mRNA methylation, thereby expanding our understanding of the post-transcriptional regulation of salt stress tolerance in rice.

## 2. Results

### 2.1. Physiological Indices of FL478 under Salt Stress

**Figure 1:**
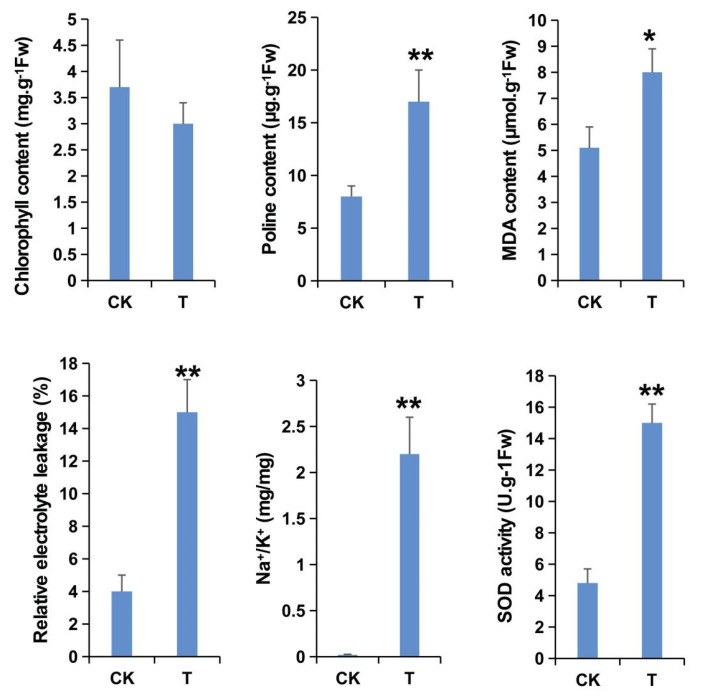
Physiological indices of FL478 plants under salt stress (T) and control (CK) conditions. Error bars indicate the standard deviation for three replicates. Asterisks indicate a significant difference between the control and salt stress conditions (* *p* < 0.05, ** *p* < 0.01; Student’s *t*-test).

The physiological traits of FL478 under control and salt stress conditions were compared. Relative to the corresponding levels under normal growth conditions, proline and malondialdehyde contents and superoxide dismutase activity increased in response to salt stress, whereas relative electrolyte leakage and Na^+^/K^+^ contents decreased. In contrast, there was no significant change in the chlorophyll content following the exposure to salt stress (Figure 1). These results indicated that FL478 grew relatively well (i.e., without any obvious salt-induced damages) during the 48 h treatment with NaCl, but there were significant changes to specific physiological indices.

### 2.2. Identification and Analysis of m^6^A Methylation Sites

**Figure 2:**
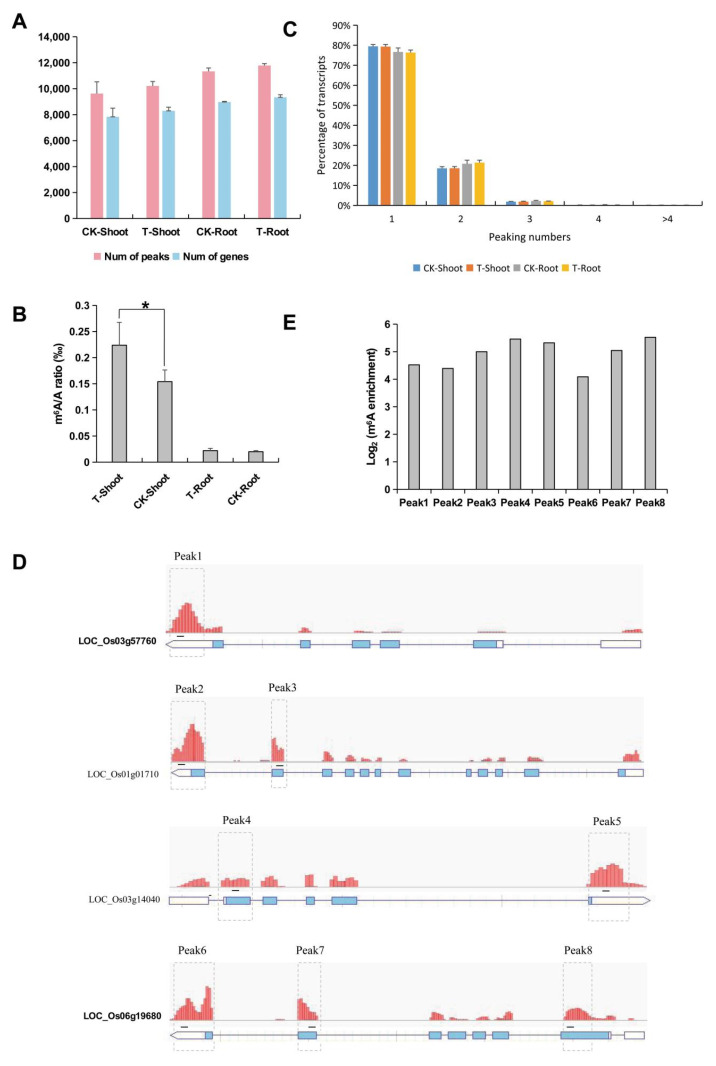
Identification and analysis of m^6^A methylation sites in rice shoots and roots under salt stress (T) and control (CK) conditions. (**A**) Number of m^6^A peaks and m^6^A-modified transcripts in rice shoots and roots under control and salt stress conditions; (**B**) m^6^A levels in the rice shoots and roots under control and salt stress conditions (* *p* < 0.05; Student’s *t*-test); (**C**) Proportions of the m^6^A-modified transcripts with one to more than four m6A peaks; (**D**) Examples of m^6^A-modified transcripts containing one m^6^A peak, two m^6^A peaks, and three m^6^A peaks. Rectangles (gray dotted lines) indicate the positions of the m^6^A peaks (peaks 1–8). Black lines indicate the positions of the amplified fragments in the following m^6^A-immunoprecipitation (IP)-qRT-PCR analysis; (**E**) Validation of the m^6^A peaks presented in panel D by m^6^A-IP-qRT-PCR.

To profile the transcriptome-wide m^6^A mRNA methylation in FL478, 12 m^6^A-immunoprecipitation (IP) and corresponding input (non-IP control) libraries were constructed and sequenced for the FL478 shoots and roots under salt stress and control conditions. After removing the adapter sequences and low-quality bases, 36.6–60.0 million clean reads were obtained per sample (Table S1). The clean reads were aligned to the rice reference genome using HISAT2 software and the MACS program was used to identify methylation sites. For each sample, the number of RNA and gene sequences with m^6^A peaks (Figure 2A) revealed that salt stress resulted in an increase in m^6^A methylation in the FL478 shoots and roots, which is basically consistent with the results obtained using the EpiQuik m^6^A RNA Methylation Quantification Kit (Figure 2B). A further analysis of the transcripts with m^6^A methylation peaks indicated that most transcripts (76.32–79.45%) had one m^6^A peak, 18.51–21.35% had two m^6^A peaks, 1.86–2.22% had three peaks, and 0.15–0.30% had four peaks; transcripts with more than four m^6^A peaks were rare (Figure 2C). Genes encoding a vesicle transporter (Os03g57760), deoxyfructose 5-phosphate reducing isomerase (Os01g01710), heat shock protein DnaJ (Os03g14040), and E3 ubiquitin ligase (Os06g19680) with one, two, two, and three methylation peaks, respectively, were selected to verify the MeRIP data by m^6^A immunoprecipitation (IP)-qRT-PCR (Figure 2D,E). As expected, these genes were substantially enriched after the mRNA immunoprecipitation with the m^6^A-specific antibody (relative to the input control) (Figure 2E). Accordingly, our m^6^A-seq data were accurate and robust.

**Figure 3:**
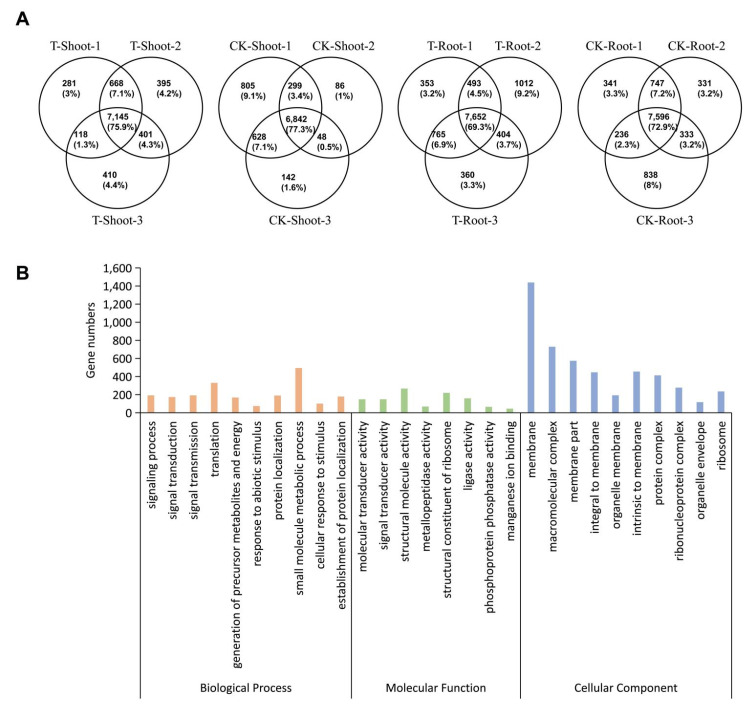
Transcriptome-wide m^6^A methylation profiles in rice shoots and roots under salt stress (T) and control (CK) conditions. (**A**) Venn diagrams presenting the overlap of m^6^A peaks identified in three independent m^6^A-seq experiments; (**B**) Gene Ontology (GO) analysis of the m^6^A-containing transcripts identified during the m^6^A-seq analysis. The functional characterization involved the annotation of GO terms from the biological process, molecular function, and cellular component categories.

Only m^6^A peaks consistently detected in all three biological replicates for each sample were designated as high-confidence m^6^A peaks. A total of 7596 and 6842 genes with high-confidence m^6^A peaks were identified in the roots and shoots, respectively, under normal growth conditions, whereas 7652 and 7145 genes with high-confidence m^6^A peaks were identified in the roots and shoots, respectively, under salt stress conditions (Figure 3A). The GO enrichment analysis of these m^6^A-containing genes revealed a potential role for m^6^A modifications in multiple signaling pathways and cellular processes (Figure 3B). Collectively, these findings demonstrated that m^6^A methylation is widespread in rice mRNA, and m^6^A-containing transcripts are related to diverse biological processes.

### 2.3. m^6^A Distribution among Sequence Motifs in the Transcriptome of FL478

**Figure 4:**
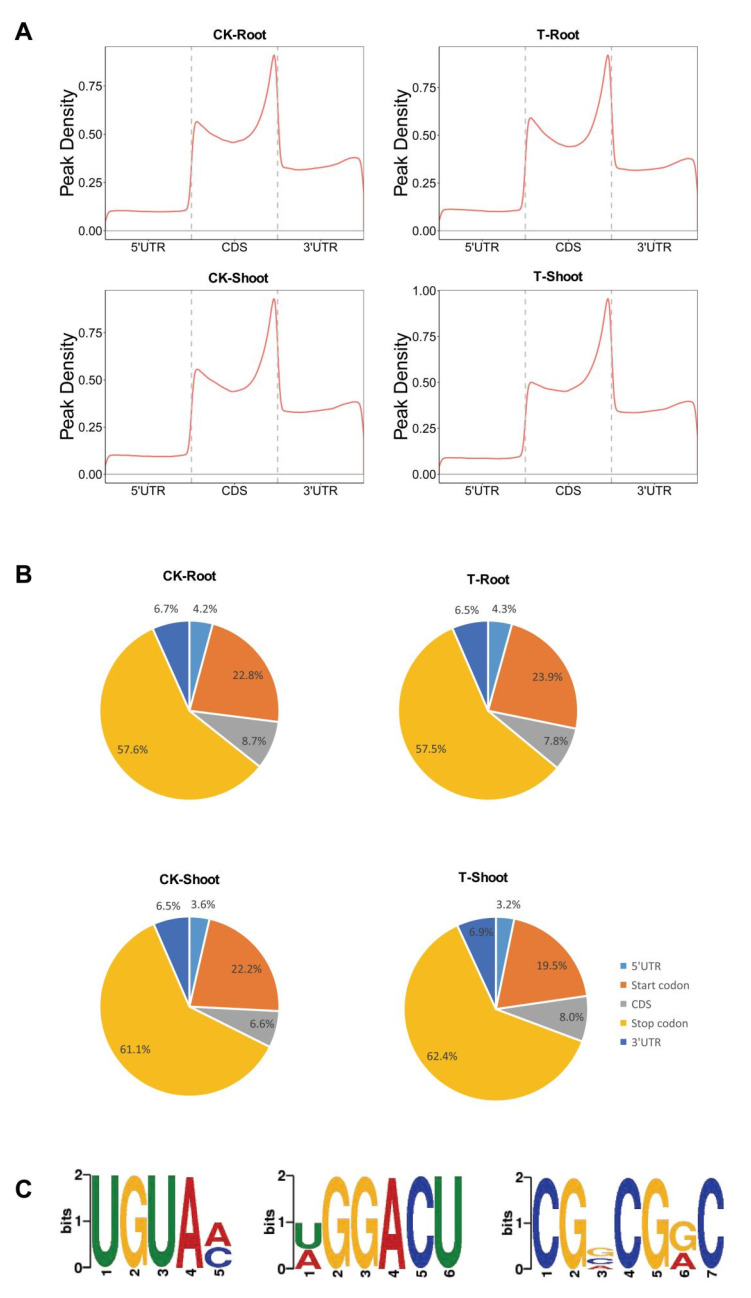
Characteristics of m^6^A localization and sequence motifs in rice shoots and roots under salt stress (T) and control (CK) conditions. (**A**) Metagenomic profiles of peak distributions along the transcripts comprising three rescaled non-overlapping segments [5′ untranslated region (UTR), coding sequence (CDS), and 3′ UTR]; (**B**) Proportions of m^6^A peaks in five non-overlapping transcript segments: transcription initiation site (TSS), 5′ UTR, CDS, termination codon, and 3′ UTR; (**C**) Sequence motifs identified within m^6^A peaks using the DREME software.

To further clarify the distribution of m^6^A peaks in the rice root and shoot transcriptomes, we mapped the metagene region methylation sites using the metaplot R package and determined the extent of the methylation in various transcript regions [5′ and 3′ UTRs and coding sequences (CDSs)] (Figure 4A). The m^6^A modification was highly enriched around the start and termination codons, which is basically in accordance with the m^6^A distribution revealed in other plant studies [7,8,16]. To confirm the distribution of m^6^A methylation within transcripts, we divided transcripts into the following five non-overlapping fragments: transcription initiation site (TSS), 5′ UTR, CDS, termination codon, and 3′ UTR. Each m^6^A RNA methylation peak was assigned to one of the five transcriptional fragments. The m^6^A peaks were significantly enriched in the stop codon fragment (100-nucleotide fragment centered on the stop codon), with 57.5–62.4% of the peaks in different samples included in this fragment (Figure 4B). Additionally, m^6^A methylation peaks were highly prevalent in the start codon region (100-nucleotide fragment centered on the start codon), which is inconsistent with the previously reported enrichment of m^6^A RNA methylation in tomato fruit and *A. thaliana* [16,23]. Overall, there were no major differences in the distribution of m^6^A peaks among the samples. Moreover, m^6^A distribution appears to differ significantly among diverse species.

To identify the sequence motifs enriched within the m^6^A peaks, we used the DREME software to visualize the sequence characteristics of m^6^A methylation regions. The following three conserved sequences were detected in these regions: UGUAM (M = A/C), which is a plant-specific conserved motif [16]; CGVCGRC (V = A/C/G, R = A/G); and DGGACU (D = A/G/U), which is similar to a conserved motif DRACH (D = A/G/U, H = A/C/U) in animals [24]. These findings reflected the conservation of sequence motifs associated with m^6^A methylation among plants and animals (Figure 4C).

### 2.4. Tissue-Specific Changes to m^6^A Methylation in Response to Salt Stress

**Figure 5:**
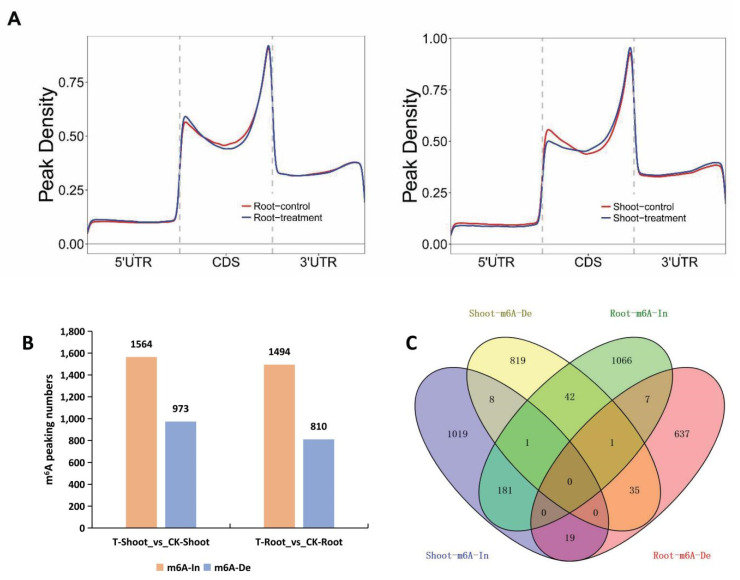
Changes in m^6^A peaks in response to salt stress. (**A**) Salt stress-induced m^6^A methylation changes in different gene regions (5′ UTR, CDS, and 3′ UTR) in the roots and shoots; (**B**) Number of genes with m^6^A methylations or demethylations in response to salt stress; (**C**) Venn diagram analysis of the genes with increased/decreased m^6^A levels (In-m^6^A/De-m^6^A) in the shoots and roots under salt stress conditions.

The extent of the m^6^A methylation of whole transcripts was plotted using the metaplot R package. We then analyzed the salt-stress-induced m^6^A methylation changes in different regions (5′ and 3′ UTRs and CDSs) in the roots and leaves. In general, transcripts in the shoots had decreased and increased m^6^A levels around the start and stop codons, respectively. In contrast, the transcripts in the roots had increased m^6^A levels around the start codon (Figure 5A).

We further screened the sites with significant changes in m^6^A methylation following the exposure to salt stress. High salinity resulted in significant increases/decreases in m^6^A methylation at 1564/973 sites and 1494/810 sites in the shoots and roots, respectively (Figure 5B). Correspondingly, there were 1228/906 and 1296/699 genes with increased/decreased m^6^A methylation in the salt-stressed FL478 shoots and roots, respectively (Tables S2–S5). A Venn diagram analysis revealed the degree of m^6^A methylation depended on the tissue for a substantial proportion of these genes under salt stress conditions (Figure 5C). Only 182 and 36 genes had the same m^6^A methylation changes in both examined tissues. Notably, some genes had the opposite m^6^A methylation changes in the shoots and roots under saline conditions. Specifically, 42 genes had increased m^6^A levels in the roots, but decreased m^6^A levels in the shoots, whereas 19 genes had increased m^6^A levels in the shoots but decreased m^6^A levels in the roots (Figure 5C, Tables S6 and S7). These results indicated that m^6^A modifications in rice are largely tissue-specifically regulated in response to salt stress.

A GO enrichment analysis was performed to functionally characterize the tissue-specific genes with differential m^6^A changes induced by salt stress. The genes with increased m^6^A methylation exclusively in the shoots were assigned the following GO terms: regulation of transcription, chloroplast, developmental process, plasma membrane, response to stimulus, and oxidoreductase activity. The genes with decreased m^6^A methylation only in the shoots were annotated with the following GO terms: signal transducer activity, response to chemical stimulus, receptor activity, and response to hormone stimulus. The genes with increased m^6^A methylation exclusively in the roots were assigned the following GO terms: oxidoreductase activity, carbohydrate metabolic process, electron carrier activity, cell wall organization or biogenesis, and response to chemical stimulus. The genes with decreased m^6^A methylation only in the roots were annotated with the following GO terms: signal transducer activity, receptor activity, and ATP-dependent helicase activity (Table S8). The results of the GO enrichment analysis suggested that genes with tissue-specific m^6^A changes are functionally related to a wide range of biological processes.

### 2.5. A Unique Set of Genes with Tissue-Specific m^6^A Changes Contributed to Salt Tolerance

As mentioned above, many genes in specific functional categories had tissue-specific m^6^A levels under salt stress (Figure 5C and Table S8). The 45 genes encoding transcriptional regulators with increased m^6^A methylation levels only in salt-stressed shoots included transcription factor (TF) genes (13 ERFs, 7 WRKYs, 4 TIFYs, and 4 ZIPs) (Table S9). Additionally, 9 and 23 genes encoding proteins with oxidoreductase activity had increased m^6^A methylation levels exclusively in the shoots and roots, respectively, under salt stress. These nine genes in the shoots included *OsACO3*, *Os2ODD7*, *Os2ODD8*, *OsLOX7*, and *OsLOX8*. The 23 genes in the roots included *OsNOX5*, *OsNOX9*, and 20 peroxidase-encoding genes (Table S9). All of these genes with increased m^6^A methylation levels may be involved in the m^6^A-mediated post-transcriptional regulation of salt tolerance.

Interestingly, among the genes encoding proteins responsive to phytohormones, 8 and 13 had salt-induced increased m^6^A levels exclusively in the shoots and roots, respectively, and 9 genes had salt-induced decreased m^6^A levels in the roots only. Most of these genes are involved in auxin signaling pathways, including *OsIAA*, *OsSAUR*, *OsARF*, and *OsAUX* genes (Table S10).

### 2.6. Main Effects of m^6^A Methylation in Rice Responses to Salt Stress

On the basis of a comparative analysis of the RNA-seq data, we identified the DEGs in the shoots and roots under control and salt stress conditions. In response to the exposure to high salinity, 481 and 330 genes had up-regulated and down-regulated expression levels, respectively, in the shoots, whereas 779 and 951 genes had up-regulated and down-regulated expression levels, respectively, in the roots. To further clarify the mechanism underlying the m^6^A methylation in response to salt stress, we analyzed the expression of genes homologous to *A. thaliana* writer and eraser genes. An examination of the corresponding transcriptome data indicated that compared with the control expression levels, three of the eight writer genes were differentially expressed under salt stress conditions. The expression levels of the *MT* homolog *OsMTA2* (LOC_Os02g45110) and the *VIRILIZER* homolog *OsVIRILIZER* (LOC_Os03g35340) were significantly down-regulated in the roots and shoots, respectively. In contrast, the expression of the *MT* homolog *OsMTA4* (LOC_Os10g31030) was highly up-regulated in the shoots. Moreover, three of the seven eraser genes were involved in the response to salt stress. The expression levels of the *ALKBH8* homolog *OsALKBH8* (LOC_Os11g43610) and the *ALKBH10A* homolog *OsALKBH10A* (LOC_Os05g33310) decreased significantly in the shoots, whereas the expression of the *ALKBH9A* homolog *OsALKBH9A* (LOC_Os06g04660) clearly increased in the shoots (Figure S1). Thus, some m^6^A ‘writers’ and ‘erasers’ may participate in rice responses to salt stress by regulating the changes in m^6^A levels.

We also analyzed the expression profiles of genes encoding m^6^A-binding proteins in the shoots and roots under control and salt stress conditions. Compared with the control expression levels, six *YTHDF* genes were differentially expressed in the shoots or roots in response to salt stress. The expression of two *YTHDF* genes (*ECT11* and *ECT6*) and one *YTHDF* gene (*ECT3*) decreased in the roots and shoots, respectively, whereas the expression of three *YTHDF* genes (*ECT2*, *ECT8*, and *ECT9*) increased in the shoots under saline conditions (Figure S1). Hence, methylation recognition proteins (readers) are regulated by salt stress and may mediate the responses of genes with differential salt-induced m^6^A levels by binding to m^6^A sites.

### 2.7. m^6^A Modifications Involved in the Regulation of Gene Expression in FL478 under Salt Stress Conditions

**Figure 6:**
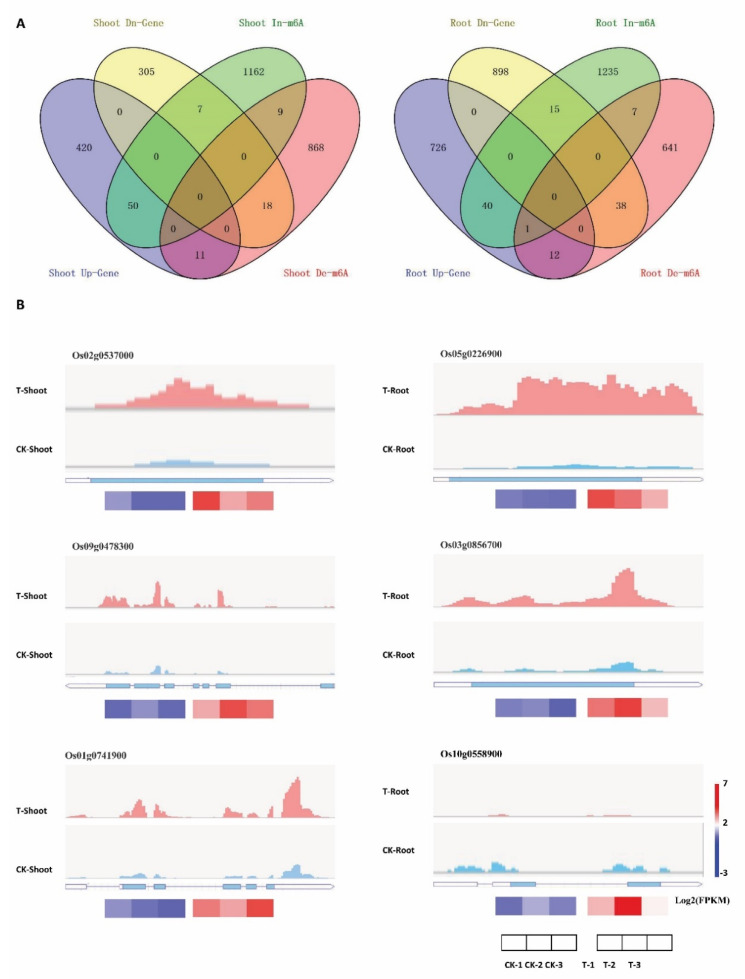
Comparative analysis of the differentially expressed genes and the genes with decreased and increased m^6^A methylation levels in the shoots and roots of FL478 plants under salt stress conditions. (**A**) Venn diagram analysis of the up-regulated (Up-gene)/down-regulated genes (Dn-gene) and the genes with increased/decreased m^6^A levels (In-m^6^A/De-m^6^A) in the shoots and roots under salt stress conditions; (**B**) Peak m^6^A levels and expression patterns of six genes in the shoots and roots under salt stress (T) and control (CK) conditions.

Recent studies on gene functions demonstrated that m^6^A mRNA modifications are important for the regulation of gene expression in plants responding to abiotic stresses [25,26]. To clarify the relationship between m^6^A modifications in mRNA and gene expression, we analyzed the DEGs with differential m^6^A methylation changes in the shoots or roots under salt stress. Of the 57 DEGs with increased m^6^A methylation in the shoots, the expression levels of 50 and 7 genes were up-regulated and down-regulated, respectively, under salt stress. Among the 29 DEGs with decreased m^6^A methylation in the shoots, the expression levels of 11 and 18 were up-regulated and down-regulated, respectively, under salt stress. The 56 DEGs with increased m^6^A methylation in the roots comprised 40 and 16 genes with up-regulated and down-regulated expression levels, respectively, under salt stress. Of the 51 DEGs with decreased m^6^A methylation in the roots, 13 and 38 had up-regulated and down-regulated expression levels, respectively, under salt stress (Figure 6A and Table S11). Genes encoding a pectinesterase inhibitor domain protein (Os02g053700), cellulose synthase (Os09g0478300), and the auxin response protein OsIAA6 (Os01g0741900) had increased m^6^A methylation and up-regulated expression levels in the shoots under salt stress (relative to the corresponding control levels) (Figure 6B). Although the exposure to salt stress resulted in increased m^6^A methylation in *RePRP1.1* (Os05g0226900) and *OsGA20ox1* (Os03g0856700) and decreased m^6^A methylation in *RL14* (Os10g0558900) in the roots, the expression levels of all these genes were up-regulated in the roots under salt stress (relative to the corresponding control levels) (Figure 6B). Therefore, m^6^A methylation was positively correlated with gene expression changes in a unique set of DEGs in FL478 under saline conditions.

A GO enrichment analysis revealed that these DEGs with differential m^6^A changes encode proteins that affect tissue development, ion transport, and stress responses. The DEGs in the shoots included *OsIAA6*, *OsNCED4*, *OsLEA24*, *OsCSLE6*, *OsMPH1*, *OsLAT4*, *OsPUP3*, and *OsSLD* (Table S11), which reportedly modulate growth and development as well as abiotic stress tolerance in rice [27,28,29,30,31,32]. The differentially m^6^A methylated DEGs in salt-stressed roots, such as *OsGA20ox1*, *OsACA6*, *OsmiR396d*, *OsPAIR3*, *OsFAD3*, *OsWAK3*, *OsWAK25*, *OsMYBR1*, *OsGORK*, and *OsHMA3*, may affect salt stress responses at the transcriptional and post-transcriptional levels.

## 3. Discussion

High salinity is a serious environmental stress that limits global crop growth and yield. Rice is highly sensitive to salt stress, especially at the seedling and reproductive stages. Several studies clarified the physiological, cellular, and molecular mechanisms mediating rice responses to salt stress [20,21,22]. However, the molecular basis of the m^6^A-mediated salt stress response in rice has not been elucidated. In the present study, we performed a high-throughput m^6^A MeRIP-seq analysis and compared the changes in m^6^A methylation and gene expression in the shoots and roots of a salt-tolerant rice genotype under normal growth and saline conditions. The results revealed the importance of m^6^A modifications on salt stress responses and provided novel insights into the molecular mechanisms regulating rice salt tolerance at the post-transcriptional level.

Earlier research confirmed that m^6^A methylation varies among tissues and is distributed unevenly on rice chromosomes [7]. In this study, we observed that m^6^A methylation peaks are enriched in the start codon region (Figure 4) in addition to being widely prevalent in the stop codon region [16,23], reflecting the diversity in the distribution patterns among species. However, our results suggest that m^6^A motifs are highly conserved in plants and animals [5,6]. We identified three m^6^A motifs, UGUAM (M = A/C), DGGACU (D = A/G/U), and CGVCGRC (V = A/C/G, R = A/G), of which UGUAM (M = A/C) and DGGACU (D = A/G/U) are conserved in plant and animals, respectively [16,24]. Whether CGVCGRC (V = A/C/G, R = A/G) is a specific motif in rice remains to be determined.

Post-transcriptional regulatory processes, including m^6^A modifications, influence plant responses to abiotic stresses. Previous studies demonstrated that salinity stress can increase the degree of m^6^A methylation in *A. thaliana* [15,16]. In the present study, the m^6^A methylation level increased substantially in the shoots and roots of the salt-tolerant FL478 rice plants under salt stress. Moreover, the salt-induced increase in m^6^A methylation in different genic regions appears to vary among tissues. We detected increased m^6^A levels around the stop and start codons in the shoots and roots, respectively, implying m^6^A methylation modulates the post-transcriptional regulation of rice responses to salt stress in a tissue-specific manner. An earlier investigation involving *A. thaliana* revealed the tissue-specific changes to the RNA structurome under salt stress conditions [18]. Tissue-specific methylation changes might affect transcript stability or translation efficiency, but their precise effects on salt stress responses must be further elucidated.

Transcriptome-wide m^6^A-seq analyses detected distinct m^6^A modifications in different plant organs or tissues [7,33]. Consistent with these findings, we observed that the m^6^A methylation of many genes was specifically differentially regulated in the salt-stressed shoots or roots. Notably, a set of TF genes, including those encoding ERF, WRKY, TIFY, and bZIP family members, had increased m^6^A methylation levels exclusively in the shoots (Table S9). Transcription factors, such as ERF and WRKY TFs, are often involved in the transcriptional regulation associated with plant responses to abiotic and biotic stresses [34,35,36]. Additionally, TIFY and bZIP TFs mediate tissue growth and stress responses [37,38]. The m^6^A methylation levels of the genes encoding these TFs increased only in the shoots, suggesting m^6^A methylation positively affects the transcriptional regulation of salt tolerance. Moreover, different subsets of genes encoding proteins with antioxidant functions had increased m^6^A methylation levels in the shoots and roots, including 20 genes encoding peroxidases in the roots (Table S9). These genes, especially the peroxidase genes, may be responsible for maintaining reactive oxygen species (ROS) homeostasis in plants under stress conditions [39]. Thus, m^6^A modifications may contribute to the ROS regulation related to rice responses to salt stress. Furthermore, phytohormones (e.g., auxin) are crucial for the coordination of biological processes mediating plant development and stress responses [40]. Genes encoding auxin-responsive proteins (OsIAAs, OsSAURs, and OsAUXs) and auxin-responsive factors (OsARFs) in rice reportedly affect shoot and root growth as well as stress responses in a tissue-specific or developmental stage-specific manner [41]. The m^6^A levels of these gene family members increased or decreased significantly in the shoots or roots (Table S10), implying m^6^A modifications actively contribute to the tissue-specific regulation of salinity stress responses. These results suggest that m^6^A methylation helps regulate the transcription, redox homeostasis, and phytohormone contents associated with rice plant responses to high salinity.

The core components of the m^6^A system are the genes encoding methyltransferases (writers, MT), demethylases (erasers), and m^6^A-binding proteins (readers) [4,42,43,44,45,46,47]. Several of these genes were identified in *A. thaliana* and rice on the basis of an analysis of animal homologous genes [10,48]. In the current study, the expression levels of some of the writer and eraser genes were differentially regulated in the shoots and roots under salt stress. For example, *OsMTA4* expression was up-regulated in the shoots, whereas the *OsMTA2* and *OsVIRILIZER* expression levels were down-regulated in the roots and shoots, respectively, under salt stress. In response to the salt stress treatment, the expression levels of the eraser-encoding genes *OsALKBH8* and *OsALKBH10* were down-regulated in the shoots, but the expression of another eraser gene was up-regulated in the shoots. Accordingly, the expression level of a single writer or eraser gene was not directly related to the degree of m^6^A methylation in the shoots and roots determined using the EpiQuik m^6^A kit (Figure 2B) and by the m^6^A-IP-seq analysis. Accumulating evidence obtained from mammalian studies has indicated that the contrasting effects of m^6^A modifications on the regulation of gene expression are due to different ‘reader’ proteins [49,50]. In *A. thaliana*, *YTHD05*, *YTHD06*, and *YTHD07* expression levels are up-regulated by heat, cold, hypoxia, or flooding stress, whereas *YTHD10* expression is inhibited by cold, drought, salt, or osmotic stress [10]. In this study, compared with the control levels, 6 of 12 *YTHDF* genes were differentially expressed in the shoots or roots under salt stress. The m^6^A readers are more responsive to abiotic stress than the writers and erasers, implying that the recognition of m^6^A methylation marks is more important than the introduction or removal of m^6^A methylation marks during plant adaptations to stress [10]. How different m^6^A marks are recognized by specific reader proteins and how this interaction contributes to the regulation of target gene expression in response to different environmental stimuli must be determined.

Several studies revealed that m^6^A modifications are important for salt stress responses because of the resulting increases in mRNA abundance and the stability of stress-responsive genes in *A. thaliana* [15]. In the present study, we identified a unique set of DEGs with increased or decreased m^6^A methylation levels in the shoots or roots under salt stress, suggesting that m^6^A is not involved in large-scale transcriptome-level changes in response to environmental stimuli. Among the DEGs in the shoots, *OsIAA6*, *OsNCED4*, *OsPSY3*, *OsGA20ox1*, and *OsLEA24* are related to phytohormone signaling pathways and stress responses [27,28,51], whereas *OsCSLE6*, *OsSLD*, *OsPUP3*, and *OsMPH1* influence tissue growth and development [29,30,31,32]. Several of the DEGs in the roots, including *OsGORK*, *OsHMA3*, *OsHAK4*, and *OsPIP2-4*, are related to ion transport [52,53,54,55]. The m^6^A methylation levels of these DEGs were similarly increased or decreased in rice plants exposed to salt stress, implying that m^6^A modifications are involved in the coordinated regulation of the gene expression related to plant growth, stress responses, and ion transport under saline conditions. Although we detected a positive correlation between gene expression and m^6^A methylation changes (e.g., the m^6^A methylation level of most of the up-regulated genes increased in FL478 under salt stress), how m^6^A methylation contributes to the changes in gene expression during plant adaptations to salt stress will need to be further clarified.

## 4. Materials and Methods

### 4.1. Plant Materials and Growth Conditions

A salinity-tolerant genotype (FL478) was used in this study. The surfaces of FL478 seeds were disinfected by soaking in 0.3% sodium hypochlorite. The seeds were then immersed in water for approximately 3 days at 28 °C until they germinated, after which they were maintained in water (pH 5.5) for 5 days. The seedlings were subsequently cultured in Yoshida nutrient solution under the following conditions: 16 h light (28 °C)/8 h dark (26 °C) cycle, with a light intensity of 600 μmol × m^−2^ × s^−1^ [56,57]. Seedlings at the four-leaf stage were treated with 120 mM NaCl for 48 h and then the shoots and roots were collected for analyses of physiological indices and m^6^A mRNA methylation.

### 4.2. Analysis of Physiological Indices

Leaves were collected from FL478 seedlings exhibiting similar growth potential under the same environmental conditions. The malondialdehyde and proline contents were measured as previously described by Song et al. [58] and Bates et al. [59]. Superoxide dismutase activity was estimated as described by Ouyang et al., with minor modifications [60]. The chlorophyll content was calculated according to the Arnon formula [61], with equal amounts of anhydrous ethanol and acetone used as the extraction solution. Relative electrolyte leakage was measured as follows. Leaves (5 cm × 5 cm) were thoroughly rinsed with deionized water to remove dirt particles from the surface and then fully immersed in deionized water at room temperature for 12 h. The conductivity was measured and recorded as R1. The leaves were maintained in boiling water for 30 min and then cooled to room temperature. The conductivity was measured and recorded as R2. Relative conductivity was calculated as R1/R2 × 100%. Potassium and sodium ion contents were analyzed using a standard curve prepared before the experiment. The dry weight of each sample was determined before the sample was added to a 50 mL tube containing 100 mM acetic acid solution. The tube was placed in a water bath shaker set at 90 °C for 2 h to extract potassium and sodium ions. The supernatant was analyzed three times using an atomic absorption spectrometer.

### 4.3. RNA Sequencing (RNA-Seq) and Data Analysis

A high-throughput RNA-seq analysis was performed by Cloud-Seq Biotech Inc. (Shanghai, China). Briefly, rRNA was removed from the extracted total RNA using the NEBNext rRNA Depletion Kit (New England Biolabs, Inc., Ipswich, MA, USA). The RNA-seq libraries were constructed using the NEBNext^®^ Ultra™ II Directional RNA Library Prep Kit (New England Biolabs, Inc., Ipswich, MA, USA). The qualitative and quantitative analyses of the libraries were performed using the 2100 Bioanalyzer (Agilent Technologies, Inc., Santa Clara, CA, USA). The libraries were sequenced on an Illumina NovaSeq 6000 instrument (Illumina, Inc., Santiago, CA, USA). The quality of the generated 150 bp paired-end reads was evaluated on the basis of a Q score of 30 (Q30). After trimming the 3′ adapters and eliminating the low-quality reads using the Cutadapt software (v1.9.3) [62] with the cutoff -m 20, -q 15, the retained high-quality clean reads were aligned to the rice reference genome (IRGSP-1.0) using HISAT2 (v2.0.4) [63]. The HTSeq software (v0.9.1) with default settings was used to obtain the raw count [64] and edgeR was used for normalizing data by TMM method. The differentially expressed genes (DEGs) were identified according to the *p*-value and expression-level fold-change (*p*-value < 0.05 and fold change > 2) [65]. The DEGs were functionally characterized by conducting a Gene Ontology (GO) and pathway enrichment analysis.

### 4.4. MeRIP-Seq Analysis

The m^6^A MeRIP-seq analysis was performed by Cloud-Seq Biotech Inc. (Shanghai, China). Briefly, m^6^A RNA immunoprecipitation was completed using the GenSeq™ m^6^A RNA IP Kit (GenSeq Inc., Shanghai, China). The input sample without immunoprecipitation and the m^6^A IP samples were used for generating the RNA-seq library with the NEBNext^®^ Ultra II Directional RNA Library Prep Kit (New England Biolabs, Inc., Ipswich, MA, USA). The library quality was evaluated using the 2100 Bioanalyzer (Agilent Technologies, Inc., Santa Clara, CA, USA) and then the library was sequenced on the Illumina NovaSeq 6000 instrument (Illumina, Inc., Santiago, CA, USA) to produce 150 bp paired-end reads. Read quality was controlled on the basis of Q30. After removing 3′ adapters and low-quality reads using the Cutadapt software (v1.9.3) [62] with the cutoff -m 20, -q 15, the clean reads were aligned to the reference genome using the HISAT2 software (v2.0.4) [63] with default settings. Methylated RNA sites (peaks) were identified using the MACS program with a cutoff of *p* < 0.00001. Differentially methylated sites were identified with diffReps with a cutoff of *p* < 0.00001 and fold change > 2. The RNA sites detected by both programs that overlapped mRNA sequences were identified and filtered using home-made scripts. The differentially methylated protein-coding genes underwent a GO and pathway enrichment analysis. Genes that were simultaneously differentially expressed and m^6^A methylated (or demethylated) in response to salt stress were included in a correlation analysis of MeRIP and RNA-seq data.

### 4.5. Analysis of MeRIP-Seq Data by Quantitative Real-Time (qRT)-PCR

The MeRIP data generated using the GenSeq^®^ m^6^A MeRIP kit (Cloud-Seq Biotech Inc., China) were analyzed by conducting a qRT-PCR analysis using SYBR^®^ Premix Ex Taq™ II (TaKaRa, Kyoto, Japan) and the Applied Biosystems^®^ 7500 thermocycler (Thermo Fisher Scientific, Waltham, MA, USA). The relative expression level of each gene was calculated according to the 2^−ΔΔCt^ method [66]. Standard deviations were calculated on the basis of three technical replicates (*n* = 6). The *OsUBQ5* gene was used as an internal control to normalize target gene expression levels.

## 5. Conclusions

Our genome-wide m^6^A methylation profiling demonstrated that salt stress enhances m^6^A methylation in rice in a distinct tissue-specific manner. The m^6^A methylation sites were largely differentially regulated in the shoots and roots in response to salt stress. The GO enrichment analysis of the genes with m^6^A methylation changes induced by salt stress revealed that m^6^A modifications likely mediate salt tolerance by regulating transcription, ROS homeostasis, and auxin signaling in a tissue-specific manner. A comparative analysis of m^6^A methylation and gene expression changes following an exposure to salt stress indicated m^6^A modifications synergistically regulate the gene expression associated with plant growth, stress responses, and ion transport under salt stress conditions.

## Data Availability

The original data presented in the study are included in the article and Supplementary Materials. Further inquiries can be directed to the corresponding authors.

## Supplementary Materials

The following supporting information can be downloaded at: https://www.mdpi.com/article/10.3390/ijms23042091/s1.
