# Supplementary material for: Global N6-Methyladenosine Profiling Revealed the Tissue-Specific Epitranscriptomic Regulation of Rice Responses to Salt Stress
Source: Int J Mol Sci. 2022 Feb 14;23(4):2091. doi: 10.3390/ijms23042091 (PMC8875919; doi:10.3390/ijms23042091)
Supplement: Supplementary file 1 [file ijms-23-02091-s001.zip › Figure S1.pdf]

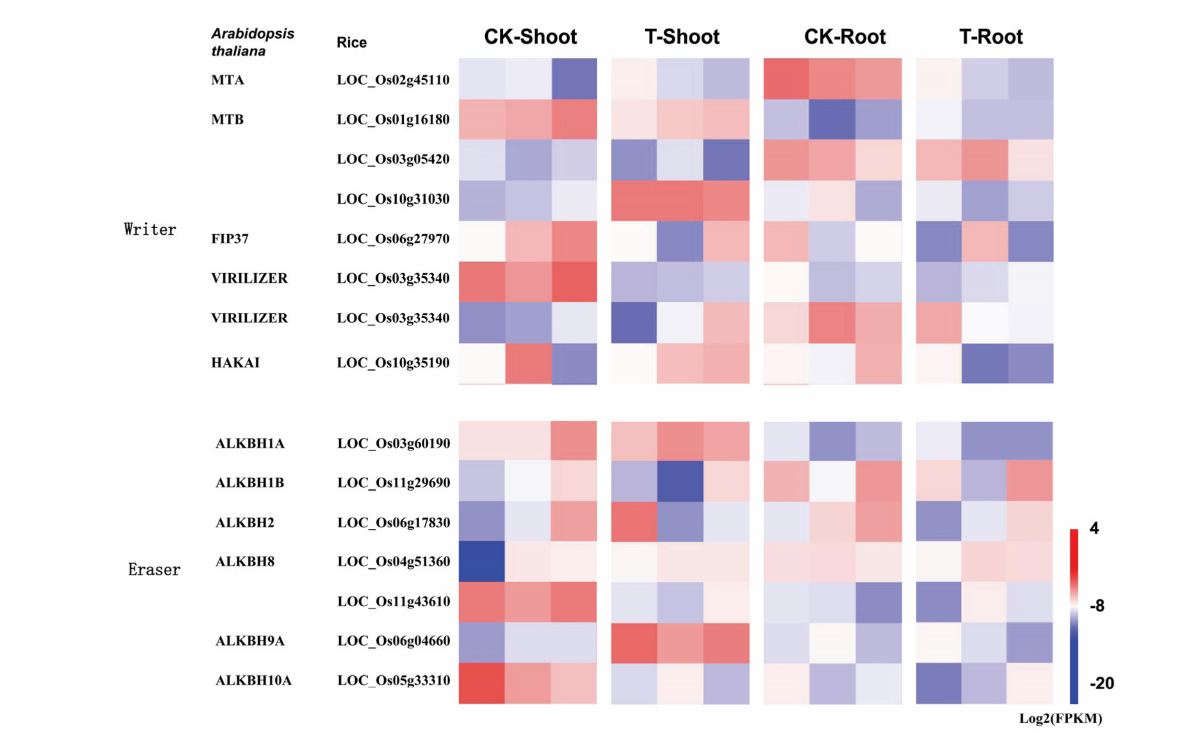

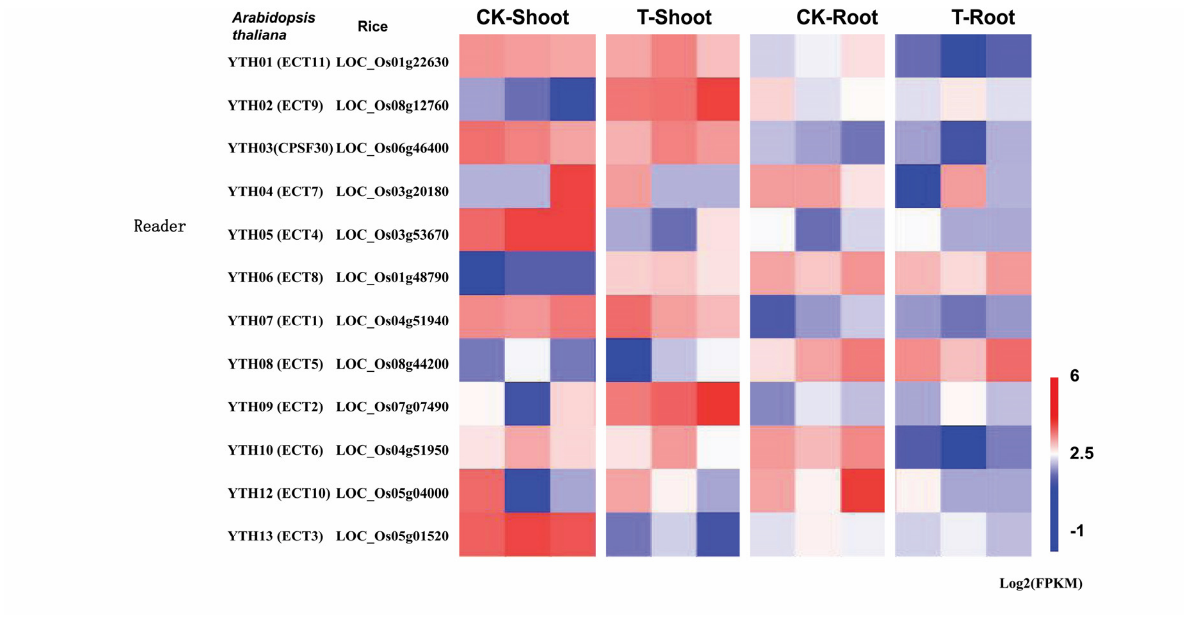

Figure S1. The expression profiling of the main component of m6A methylation in shoots and roots of FL478 under salt stress.
